# *Diplotaxis muralis* as an Emerging Food Crop: Chemical Composition, Nutritional Profile and Antioxidant Activities

**DOI:** 10.3390/plants14060844

**Published:** 2025-03-08

**Authors:** Sandrine Ressurreição, Lígia Salgueiro, Artur Figueirinha

**Affiliations:** 1 University of Coimbra, Faculty of Pharmacy 3000-548 Coimbra, Portugal; sandrine@esac.pt (S.R.); ligia@ff.uc.pt (L.S.); 2 Polytechnic of Coimbra, Coimbra Agriculture School, 3045-601 Coimbra, Portugal; 3 Chemical Engineering and Renewable Resources for Sustainability (CERES), Department of Chemical Engineering, University of Coimbra, 3030-790 Coimbra, Portugal; 4 Associated Laboratory for Green Chemistry (LAQV) of the Network of Chemistry and Technology (REQUIMTE), University of Coimbra, 3000-548 Coimbra, Portugal

**Keywords:** natural resources, nutritional potential, health benefits, secondary metabolites, bioactivities

## Abstract

*Diplotaxis muralis* (L.) DC (Brassicaceae) is an edible plant commonly used in Mediterranean diets. This study investigates its nutritional composition, secondary metabolites, and antioxidant activity. The results show that this plant is rich in fibre and essential minerals. Analysis of amino acids shows a diverse profile, with glutamic acid and aspartic acid being the most abundant. Regarding fatty acids, α-linolenic acid was identified as predominant. Importantly, levels of toxic metals such as cadmium, lead, and mercury were found to be within established safety limits, confirming the plant’s suitability for consumption. A leaf decoction using 80% methanol exhibited the highest concentrations of total phenolic compounds (68.36 mg eq. gallic acid g^−1^), total flavonoids (3.50 mg eq. quercetin g^−1^), and antioxidant activity (IC₅₀ of 78.87 µg mL^−1^ for ABTS, 392.95 µg mL^−1^ for DPPH, and a FRAP value of 731.20 µmol Fe(II) g^−1^). HPLC-PDA-ESI-MSⁿ characterization identified flavonols as the main polyphenols. Additionally, several glucosinolates were identified. These compounds, along with their hydrolysis products, not only contribute to the health benefits of *D. muralis*, but also impart its distinctive pungent and spicy notes, playing a crucial role in shaping its unique sensory profile. These findings highlight the contribution of phenolic compounds and glucosinolates to the health benefits of *D. muralis*, reinforcing its potential as a promising plant for the development of new functional foods.

## 1. Introduction

The growing global population is placing increasing demands on agriculture to ensure sufficient food supply. This has led to innovations in agricultural practices, value chains, and products aimed at enhancing food availability and sustainability [1]. These innovations include the introduction of non-conventional plant-based foods, which offer a sustainable alternative by alleviating resource pressure and diversifying food sources [2]. Rich in nutrients and more sustainably cultivated, these foods are critical for future food security [3]. They not only satisfy the rising food demand but also reduce the ecological footprint of agricultural production. Thus, transitioning to a sustainable food system that includes non-conventional plant-based foods is crucial for ensuring access to nutritious and responsibly produced food for everyone [3,4].

The Brassicaceae family includes numerous species across various genera, many of which play a significant role in human nutrition. Among the genera that have garnered increased attention in recent years is *Diplotaxis*, comprising approximately 40 species [5]. Notably, *D. tenuifolia* stands out as it is widely cultivated and consumed across different parts of the world due to its distinctive flavour and nutritional properties [6]. However, other species within the genus, such as *D. muralis*, have also attracted growing interest, whether for their potential as a food source or for other applications.

**Figure 1:**
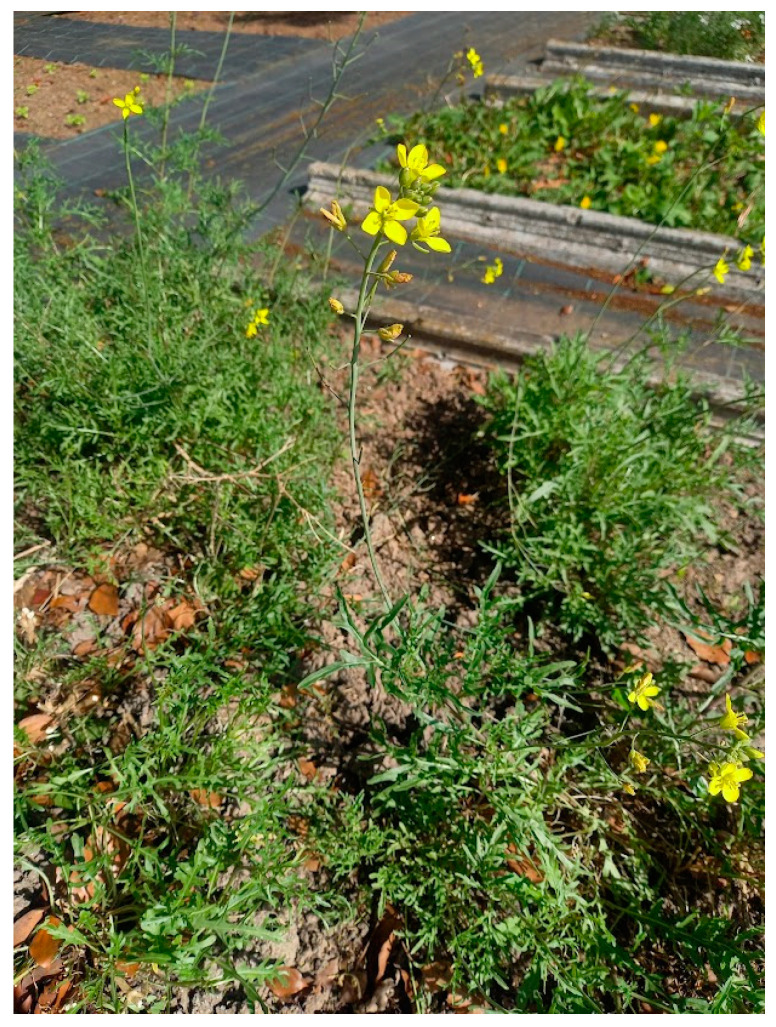
*Diplotaxis muralis* (L.) DC.

*Diplotaxis muralis* (L.) DC (Figure 1), commonly known as annual wall rocket or mustard rocket, is native to the Mediterranean region, naturally occurring in mainland Portugal, and introduced in the Azores [5]. This edible plant is widely valued for its culinary versatility. Its leaves can be consumed raw in fresh salads, offering a crisp and refreshing texture, or used as an ingredient in a variety of dishes, such as pizzas, sandwiches, and soups. They can also be processed, such as dried or frozen, to extend their shelf life and broaden their applications in different culinary contexts [5]. *D. muralis* exhibits a spicy and pungent flavour profile, characteristic of the edible plants of this genus, which are frequently used in cooking. This trait is particularly pronounced in this species, making it especially appealing. The unique sensory characteristics of *D. muralis* are largely attributed to its glucosinolate content and the products derived from their hydrolysis [7]. Glucosinolates contribute to the plant’s characteristic bitter and spicy notes, while the release of volatile isothiocyanates during hydrolysis imparts a sharp, aromatic intensity [8]. The flavour profile of *D. muralis* leaves evolves significantly with their stage of development. Younger leaves are tender and have a mild, slightly peppery taste, making them ideal for raw preparations like salads or as delicate garnishes [8]. In contrast, mature leaves present a more intense, spicy, and pungent flavour, which enhances their appeal in robust dishes like soups, sauces, or as a garnish for hot meals. This flavour contrast across developmental stages offers chefs and consumers a versatile range of options to suit various tastes and applications, a unique combination that is highly appreciated and underscores the species’ culinary and commercial potential [8].

However, there are limited studies on the chemical composition of *D. muralis*, particularly regarding its nutritional profile and secondary metabolite composition. Therefore, the objective of this study is to characterize the nutritional content and secondary metabolites of *D. muralis*, as well as to explore its antioxidant activities for both health and dietary applications. This research aims to provide a deeper understanding of the plant’s properties and its potential health-related benefits.

## 2. Results and Discussion

### 2.1. Nutritional Composition

The leaves of *D. muralis* were subjected to a comprehensive nutritional analysis, with the detailed results presented in Table 1. Although there are several studies on the nutritional properties of plants in the *Diplotaxis* genus, no specific studies have been found regarding dietary fibre in these species.

This study, therefore, focuses on the detailed characterisation of the different fibre fractions present in the leaves of *D. muralis*, with an emphasis on dietary fibre, including its total, soluble, and insoluble fractions. The analysis of these fractions is crucial for understanding the nutritional potential of this plant and its possible food applications [5,6,9,10,11]. The dietary fibre content of *D. muralis* is 29.52% in dry matter, which falls within the range observed for different varieties of *Brassica oleracea*, where the dietary fibre content varies from 18.81% to 34.83% in dry matter, according to the PortFIR database (https://jspapp.test.insa.foodcase-services.com/, accessed on 14 January 2025) [12]. Thus, the fibre content of *D. muralis* is relatively high compared to most varieties of *Brassica oleracea*. Dietary fibre has been widely recognised for its physiological benefits to health. Insoluble fibre, which makes up the majority of dietary fibre, plays a crucial role in promoting regular bowel movements and preventing constipation by increasing stool volume and facilitating its passage through the digestive tract [13]. However, this fibre is not easily fermented by intestinal bacteria or is fermented slowly. In contrast, soluble fibre, although present in smaller quantities, positively impacts gut health. It is quickly metabolised by gut bacteria, which beneficially influences the abundance and diversity of the gut microbiota. Additionally, soluble fibre is metabolised into beneficial products, such as short-chain fatty acids (SCFAs), which provide various health advantages, including reducing the risk of gastrointestinal diseases [13].

*D. muralis* leaves contain 2.27 g 100 g^−1^ of protein, with a diverse amino acid composition, presented in Table 2. The most abundant amino acids include glutamic acid and aspartic acid, followed by leucine and proline. Other notable amino acids are alanine, lysine and phenylalanine. The leaves also provide smaller amounts of essential amino acids, such as histidine and methionine. This amino acid profile suggests that *D. muralis* leaves could be a valuable addition to a balanced diet.

Although studies on the amino acid profile of plants within the *Diplotaxis* genus are lacking, research on other Brassicaceae like broccoli (*Brassica oleracea* var. *italica*), kale (*Brassica oleracea* var. *acephala*), turnip (*Brassica rapa*), and arugula (*Eruca sativa*) shows that glutamic acid and aspartic acid are the most abundant amino acids, a pattern also seen in *D. muralis* [14,15,16,17].

Following the analysis of the amino acid profile, the mineral content is presented in Table 3.

According to Regulation (EU) No 1169/2011 of the European Parliament and the Council of 25 October 2011, mineral nutrient content is considered significant when it exceeds 15% of the Nutrient Reference Value (NRV) [18]. *D. muralis* contains notable levels of essential minerals, including calcium, potassium, phosphorus, magnesium, iron, manganese, and chromium. However, there are no specific studies on the mineral composition of *D. muralis* leaves for direct comparison of results. Nevertheless, *D. muralis* shows higher concentrations of calcium, phosphorus, and ash, while *D. tenuifolia* contains higher levels of potassium and iron. Both plants are valuable sources of essential nutrients with complementary mineral profiles [5,6,11].

The fatty acid profile analysis of *D. muralis* was also carried out. The results are presented in Table 4.

The fatty acid composition analysis of *D. muralis* reveals that α-linolenic acid is the most abundant fatty acid, constituting 40.58%. In comparison, the lipid composition of *D. simplex* leaves has been studied, showing α-linolenic acid as the most abundant fatty acid, constituting 25.4%. Additionally, *D. simplex* contains palmitic acid (13.2%), oleic acid (7.7%), linoleic acid (4.4%), and ethyl linoleate (14.4%) [5,10].

### 2.2. Food Safety

Brassicaceae plants are known for their tendency to accumulate heavy metals, a trait that makes them valuable for the phytoremediation of contaminated soils. However, this same ability necessitates careful monitoring, as the accumulation of heavy metals can also pose potential health risks [5,19]. To ensure safe cultivation, the soil and irrigation water were analysed prior to planting (Table A1 and Table A2) to verify compliance with the required quality standards. Additionally, comprehensive control and safety analyses were performed, including the assessment of toxic heavy metals such as cadmium, lead, and mercury in the harvested *D. muralis* leaves, to evaluate the presence and levels of these contaminants (Table 5). Commission Regulation (EU) No 2023/915 of 25 April 2023 sets the maximum limits for brassicas at 10 µg per 100 g for lead, 4 µg per 100 g for cadmium, and 10 µg per 100 g for mercury, applicable to food supplements and salt [20]. The *D. muralis* samples were found to be below these established limits. These safety analyses are crucial to ensure that the cultivated plants do not pose any health risks, thus guaranteeing the food safety of the final product. Rigorous control of environmental parameters and contaminants is essential not only for the validity of the results but also for safeguarding the safety of future applications of *D. muralis* in food and nutritional contexts.

### 2.3. Secondary Metabolites

Phenolic compounds and glucosinolates are abundant and characteristic of the genus, exhibiting a broad spectrum of biological activities such as antioxidant, anti-inflammatory, antibacterial, hypoglycaemic, hypolipidemic, cytotoxic, and antiproliferative activities [5]. Variations in these biological activities can be attributed to several factors, including extraction conditions, which significantly influence the chemical composition of the extracts. In order to extract a broader diversity of these compounds, *D. muralis* leaves were subjected to various extraction methods: decoction in 80% methanol, maceration in 100% and 50% ethanol, and maceration and infusion in 100% water. The extraction yields were 1.2, 1.4, 2.2, 4.7, and 5.8%, respectively. The total phenolic and flavonoid content was quantified, and the results of this analysis are presented in Table 6.

The extraction methods used had a significant impact on the levels of total phenolic compounds and flavonoids in the *D. muralis* extracts. Among the methods used, decoction in methanol (80%) produced the highest concentrations of phenolic compounds, 68.36 mg of gallic acid equivalents (GAE) g^−1^, and flavonoids, 3.50 mg of quercetin equivalents (QE) g^−1^. Although no previous studies have examined the phenolic and flavonoid content of this species, these findings align with existing research on other *Diplotaxis* species, which have demonstrated varying levels of phenolic and flavonoid content. For example, methanolic extracts from the leaves of *D. simplex* and *D. harra* had lower total phenolic contents, at 4.79 mg GAE g^−1^ and 5.47 mg GAE g^−1^, respectively [21]. The ethanolic extract from the pre-flowering aerial parts of *D. harra* exhibited a higher concentration of phenolic compounds, with 80.43 mg GAE g^−1^, as well as a higher concentration of flavonoids, with 54 mg QE g^−1^ [22].

HPLC-PDA-ESI-MS^n^ analysis was performed on D. *muralis* leaves after decoction in methanol (80%); this method was selected for its high concentrations of total phenolic compounds and flavonoids. This analysis enabled the identification of specific compounds in the extract, with the identified compounds presented in Table 7.

#### 2.3.1. Glucosinolates

Peak 2 exhibited a deprotonated molecular ion [M−H]^−^ at *m*/*z* 436, along with characteristic fragment ions of glucosinolates at m/z 274, 259, 195, 96, and 74, which result from cleavages on either side of the thioether bond. A signal at *m*/*z* 372, corresponding to the neutral loss of methanesulfenic acid (CH_3_SOH, 64 Da), serves as a diagnostic fragment for all methylsulfinyl glucosinolates. Additionally, the loss of a methyl group from the side chain produced a fragment ion at *m*/*z* 421. The ion at *m*/*z* 178 corresponds to the loss of a thioglucose moiety, while the neutral loss of 242 Da results in a fragment at *m*/*z* 194. Based on this fragmentation pattern, peak 2 was identified as glucoraphanin [25,26]. Peak 3 showed a pseudomolecular ion at *m*/*z* 494, generating several characteristic fragments. The ion at *m*/*z* 332 corresponds to the loss of a hexosyl group. Fragment ions at *m*/*z* 316 and *m*/*z* 298 can be explained by neutral losses of 178 Da and 196 Da, respectively, both corresponding to the loss of a d-thioglucose unit from the precursor ion at *m*/*z* 494. This fragmentation pattern is consistent with the presence of a glucosinolate containing three sulfur atoms, identified as 6-methylsulfonyl-3-oxohexyl-glucosinolate [27]. For Peak 4, a pseudomolecular ion at *m*/*z* 600 was observed, generating fragment ions at *m*/*z* 438, 420, and 404, corresponding to the neutral losses of C_6_H_10_O_5_ (162 Da), SC_6_H_10_O_5_ + H_2_O (180 Da), and C_6_H_10_O_5_ + H_2_S (196 Da), respectively. A glucosinolate with an intermolecular disulfide linkage, specifically 4-(*β*-D-glucopyranosyldisulfanyl)butyl-glucosinolate, displaying a similar fragmentation pattern in *Eruca sativa* L., was previously identified. Based on these findings, the proposed structure for compound 4 is glucopyranosyldisulfanylbutyl-glucosinolate [28]. In the case of Peak 5, a deprotonated molecular ion [M−H]^−^ was detected at *m*/*z* 420, consistent with a methylthioglucosinolate. Typical fragment ions were detected at m/z 275, 259, and 195. Additionally, the ion at *m*/*z* 178, corresponding to a neutral loss of 242 Da, was previously described. These spectral characteristics support the identification of this compound as glucoerucin [26,29]. Finally, Peak 7 presented a precursor ion at *m*/*z* 406, generating typical glucosinolate fragment ions at *m*/*z* 274, 195, 164, 96, and 74. Based on its fragmentation behaviour, this compound was tentatively identified as glucoiberverin [26,29].

Among the five glucosinolates identified, glucoraphanin and glucoerucin had already been reported in this species, while glucoiberverin, glucopyranosyldisulfanyl-butyl-glucosinolate, and 6-methylsulfonyl-3-oxohexyl-glucosinolate stand out as they have not been previously described in *D. muralis* [5]. Pungent and spicy flavours are characteristic of *D. muralis*, primarily due to the presence of isothiocyanates, which result from the hydrolysis of glucosinolates by the enzyme myrosinase. Glucoraphanin is converted into the isothiocyanate sulforaphane, which imparts a pungent, slightly bitter flavour commonly found in vegetables like kale and broccoli [41]. 6-methylsulfonyl-3-oxohexyl-glucosinolate releases an isothiocyanate with a mildly sulfurous and spicy flavour, reminiscent of mustard [41]. Glucopyranosyldisulfanyl-butyl-glucosinolate, when broken down, generates butyl isothiocyanate, which results in a pungent and spicy flavour, similar to that of radish or mustard [41]. Glucoerucin produces erucin, an isothiocyanate with a spicy and bitter flavour, with cabbage-like notes [7]. Finally, glucoiberverin, present in vegetables like broccoli and cabbage, yields iberverin, which also imparts a pungent flavour, similar to radish, with strong intensity [7]. These pungent and spicy flavours are characteristic of *D. muralis*, where the presence of these isothiocyanates contributes to its distinct flavour.

#### 2.3.2. Phenolic Compounds

##### Phenolic Acids

Peak 6 was identified as gentisic acid-*O*-hexoside, exhibiting a deprotonated molecular ion [M−H]^−^ at *m*/*z* 315. The major fragment ion at *m*/*z* 153 results from the loss of 162 Da, corresponding to a dehydrated hexose, while the ion at *m*/*z* 109 corresponds to the consecutive loss of a hexose and carbon dioxide ([M−H−162−44]^−^). Hydroxybenzoic acids typically exhibit a λmax between 200 and 290 nm, except for gentisic acid, whose absorbance extends up to 355 nm. The UV spectrum of this compound, with a maximum absorption at 290 nm, aligns with this hypothesis [30,31]. Peak 10 exhibited a pseudomolecular ion at *m*/*z* 385, which underwent the loss of a hexosyl moiety (−162 Da), yielding a fragment ion at *m*/*z* 223. Additional MS^2^ fragments included a signal at *m*/*z* 205, corresponding to the loss of a water molecule. Based on this fragmentation pattern, the proposed structure for compound 10 is sinapic acid-*O*-hexoside [28]. Peak 19 displayed a UV spectrum characteristic of hydroxycinnamic acids, with a maximum absorption at 326 nm. The first-order mass spectrum exhibited a pseudomolecular ion at *m*/*z* 753, followed by sequential losses of hexosyl and sinapoyl units. This fragmentation behaviour has been previously described in the literature for disinapoylgentiobiose [36,37].

This is the first time the three identified compounds have been reported in *D. muralis*. Only *D. harra* and *D. simplex* have been found to contain distinct derivatives of hydroxycinnamic and hydroxybenzoic acids [5,21,42,43].

##### Flavonols

Peaks 8, 11, 15, and 16 exhibited UV spectra consistent with quercetin. The high intensity of Band II in the UV spectrum suggests glycosylation at position 3. Peaks 8 and 11 displayed pseudomolecular ions at *m*/*z* 949 and *m*/*z* 787, respectively, with successive losses of hexosyl units, yielding a fragment ion at *m*/*z* 301. This fragmentation pattern is characteristic of glycosylated quercetin, leading to the identification of these compounds as quercetin-*O*-tetrahexoside (Peak 8) and quercetin-*O*-trihexoside (Peak 11). Peak 16 presented a similar fragmentation behaviour but included the loss of a deoxyhexosyl unit. Consequently, this compound was identified as quercetin-*O*-deoxyhexose-hexose [34]. Peak 15 showed a deprotonated molecular ion at *m*/*z* 993, with fragment ions at *m*/*z* 831 and *m*/*z* 669, resulting from the sequential loss of hexosyl residues. Additionally, a signal at *m*/*z* 463 was observed due to the loss of a sinapoyl moiety (−206 Da), followed by another hexosyl loss, yielding *m*/*z* 301. This fragmentation pattern has been previously reported in *Eruca* and *Diplotaxis* species, leading to the tentative identification of this compound as quercetin-3,4′-diglucoside-3′-(6-sinapoyl-glucoside) [35].

The UV spectra of Peaks 9, 12, and 17 exhibited Band II near 265 nm and a lower-intensity Band I between 320 and 350 nm, which is consistent with a 3-*O*-substituted flavonol monohydroxylated at ring B, likely kaempferol. Peak 9 displayed a deprotonated molecular ion at *m*/*z* 771, with fragment ions at *m*/*z* 609, *m*/*z* 447, and *m*/*z* 285, resulting from the sequential loss of hexosyl residues. This fragmentation pattern suggests that compound 9 is kaempferol-O-triglucoside. Peak 12 exhibited a pseudomolecular ion at *m*/*z* 609 and successive losses of hexosyl units, generating an aglycone fragment at *m*/*z* 285. Compared to peak 9, this compound has one fewer hexosyl substituent, suggesting its identification as kaempferol-*O*-dihexoside [34]. Peak 17 was identified as kaempferol-*O*-deoxyhexoside-hexoside, as its pseudomolecular ion at m/z 593 produced a fragment ion at *m*/*z* 285 due to the loss of 308 Da, corresponding to the combined loss of a hexosyl (162 Da) and a deoxyhexosyl (146 Da) unit [34].

Peaks 13, 14, and 18 exhibited a typical 3-*O*-substituted flavonol UV spectrum. The fragmentation pattern of these compounds was characterized by the sequential loss of hexosyl units, generating a fragment ion at *m*/*z* 315, which can be attributed to rhamnetin or isorhamnetin. An additional loss of a methyl group resulted in a fragment ion at *m*/*z* 300. Rhamnetin produces two signals: a high-intensity peak at *m*/*z* 299 and a slightly less intense peak at *m*/*z* 300, as reported [44]. In contrast, isorhamnetin predominantly undergoes CH_3_ loss, resulting in a single intense peak at *m*/*z* 300. Based on this distinction, the proposed structures for these compounds are isorhamnetin-*O*-trihexoside (Peak 13), isorhamnetin-*O*-dihexoside (Peak 14), and isorhamnetin-*O*-dihexoside (Peak 18) [34,44].

The phenolic compounds identified are primarily flavonol di-, tri- or tetra-*O*-glycosides of kaempferol, quercetin, and isorhamnetin. These types of compounds are commonly reported within the genus *Diplotaxis*, although there are currently no data available on the compositional profile of *D. muralis* leaves. The most abundant of these include quercetin-3-*O*-hexoside-dihexoside, quercetin-3,4′-diglucoside-3′-(6-sinapoyl-glucoside), and quercetin-3-*O*-deoxyhexose-hexose. No mono-*O*-glycosides were identified, which is typical for the genus, while tetra-*O*-glycosides were identified for the first time. Additionally, quercetin-3-*O*-tetrahexoside, kaempferol-*O*-triglucoside, and isorhamnetin-*O*-trihexoside are reported in this genus for the first time. Among *Diplotaxis* species, *D. tenuifolia* has been the most extensively studied, with its predominant flavonoids including quercetin 3,3′,4′-triglucoside, quercetin 3,4′-diglucoside-3′-(6-sinapoyl-glucoside), and quercetin 3-(2-sinapoyl-glucoside)-3′-(6-sinapoyl-glucoside)-4′-glucoside [5,35,45,46,47,48].

#### 2.3.3. Fatty Acids and Lipids

Peak 1 exhibited a pseudomolecular ion at *m*/*z* 333. The base peak at *m*/*z* 153 corresponds to the loss of the inositol moiety. Other characteristic fragment ions included *m*/*z* 241 [M−glycerol]^−^, *m*/*z* 153 [M−inositol]^−^, and *m*/*z* 79.1 [HPO_3_]^−^. Based on this fragmentation pattern, compound 1 was identified as glycerophosphoinositol [23,24]. Peak 20 showed a deprotonated molecular ion [M−H]^−^ at *m*/*z* 327. The major fragment ions were observed at *m*/*z* 309, 291, 229, and 211, which, according to previous reports, are characteristic of an oxylipin compound, oxo-dihydroxy-octadecenoic acid (DHODE) [38]. For Peak 22, a pseudomolecular ion at *m*/*z* 309 was observed displaying a fragmentation pattern identical to that of compound 20, suggesting the presence of another oxylipin with one fewer hydroxyl group. Thus, it was identified as 8-hydroxy-9-oxo-octadecanoic acid [39]. Peak 24 was identified as 9-hydroxy-octadecatrienoic acid, as it exhibited the same pseudomolecular ion and fragmentation pattern as a previously reported compound [38]. Peaks 21 and 23 displayed the same pseudomolecular ion at *m*/*z* 311 and an identical fragmentation profile. The fragment ion at *m*/*z* 293 corresponds to the loss of a water molecule. Based on the previous literature, these compounds were tentatively identified as arachidic acid [38]. Finally, Peak 25 exhibited a pseudomolecular ion [M−H]^−^ at *m*/*z* 675 and a spectral profile consistent with that of a hexosyl lipid, specifically *O*-(hexosyl-hexosyl)-*O*-linolenoyl-glycerol, as previously reported [40].

This is the first time the six identified compounds have been reported in *D. muralis*. In the *Diplotaxis* genus, arachidic acid has only been identified in the flowers of *D. simplex*, and other oxylipins have been reported in *D. erucoides* [5,10,34].

### 2.4. Antioxidant Activity

Quercetin, kaempferol, and isorhamnetin *O*-glycosides, along with glucosinolates, are abundant and characteristic compounds of the *Diplotaxis* genus, recognized for their wide range of biological activities. Considering the presence of these types of compounds in *D. muralis*, we assessed its antioxidant potential to explore possible health benefits. The antioxidant activity of the extracts was evaluated through ABTS, DPPH, and FRAP assays, with the results summarized in Table 8.

The most active extract of *D. muralis* was the methanol decoction (80%), which exhibited an IC50 of 78.87 µg mL^−^^1^ for ABTS, 392.95 µg mL^−^^1^ for DPPH, and a value of 731.20 µmol eq. Fe(II) g^−^^1^ for the FRAP assay. These results align with the high levels of phenolic and flavonoid compounds observed in this extract, indicating its strong antioxidant activity. The ABTS test, which involves both polar and apolar compounds, showed the lowest IC_50_ value, suggesting the dominant role of phenolic compounds in the antioxidant activity. In contrast, the DPPH test revealed a greater contribution from apolar compounds, although the IC_50_ was higher compared to ABTS, reflecting a less efficient antioxidant activity. The FRAP assay confirmed the presence of compounds with high reducing activity, likely phenolic compounds, contributing to the neutralisation of free radicals.

Although there are no prior studies on the antioxidant activity of *D. muralis*, other species from the same genus have been investigated, namely *D. harra*, *D. simplex* and *D. erucoides* [22,34,49]. In the ABTS assay, the IC_50_ values for the extracts ranged from 97.87 to 920 µg mL^−^^1^, with the ethanolic extracts from *D. erucoides* showing the best antioxidant activity. However, *D. muralis* methanolic extract demonstrated even better results with an IC_50_ of 78.87 µg mL^−^^1^. In the DPPH assay, the IC50 values ranged from 135.13 to 5470 µg mL^−^^1^, with *D. muralis* showing a value within this range. In the FRAP assay, the ethanol extract of *D. erucoides* obtained a value of 24.42 μM Fe(II) g^−^^1^, a value lower than that observed for the extracts of *D. muralis* [34]. The observed differences in antioxidant activity can be attributed to variations in the extraction processes, including differences in solvent type, extraction time, and plant quantity. Additionally, variations in antioxidant methodologies may also play a role. The distinct chemical profiles of the extracts could explain the differences in activity. However, for most species and extracts, studies on their chemical composition have not been conducted.

Phenolic compounds and flavonols, such as quercetin and kaempferol, enhance antioxidant defence mechanisms by neutralizing free radicals and upregulating endogenous antioxidant enzymes, such as superoxide dismutase, catalase, and glutathione peroxidase, thereby strengthening cellular defences against oxidative damage [50,51]. Similarly, glucosinolates and their hydrolysis products, along with phenolic compounds and fatty acids, exhibit antioxidant activity. Isothiocyanates, derived from glucosinolate hydrolysis, scavenge reactive oxygen species (ROS) and modulate cellular redox balance, contributing to anti-inflammatory effects by inhibiting pro-inflammatory pathways such as NF-κB pathway signalling [52,53]. Sulforaphane, the isothiocyanate resulting from the hydrolysis of glucoraphanin, has multiple biomedical applications and is recognized as an important source of chemopreventive and anticancer agents [54,55]. Similarly, erucin, the isothiocyanate derived from glucoerucin, although less studied, also exhibits these properties [56,57]. Additionally, polyunsaturated fatty acids, particularly α-linolenic acid, exert both antioxidant and anti-inflammatory properties by modulating lipid peroxidation and inflammatory mediators such as prostaglandins and cytokines [58,59]. The synergistic interaction between these bioactive compounds not only reinforces the antioxidant potential of *D. muralis* but also suggests its potential relevance in inflammation-related diseases.

## 3. Material and Methods

### 3.1. Plant Material

*D. muralis* (L.) DC. seeds were obtained from various specimens of the Living Showcase of the Collection of Aromatic, Medicinal and Spice Plants at the Coimbra Agriculture School of Polytechnic of Coimbra, located at coordinates 40°12′53.4″ N 8°27′16.1″ W. The seeds were sown in cells with peat and subsequently transferred to their final location, an area of approximately 30 m^2^. After confirming that both soil and water quality met the necessary standards (Table A1 and Table A2), the *D. muralis* crop was planted. Leaf harvesting took place in June 2023, prior to the flowering stage, when the leaves were young and tender. A voucher specimen was stored in the herbarium of the Faculty of Pharmacy of the University of Coimbra. After the harvest, the colour of the leaves was assessed on both the top and bottom surfaces using a colorimeter (Chroma Meter—CR-400, Konica Minolta, Tokyo, Japan). The values were recorded in the CIE Lab colour space. The leaf colour measurements in the CIELAB colour space were L* = 36.4, a* = −11.2, and b* = 9.4, indicating a dark hue with green and yellow undertones. A portion of the harvested leaves was used for nutritional analysis, while the remaining leaves were freeze-dried for subsequent phytochemical characterization and biological assays.

### 3.2. Nutritional Composition Analysis

The methodologies outlined by the Association of Official Analytical Chemists [60] were employed to determine the chemical properties of *D. muralis* leaves: moisture content (AOAC method 930.04), ash content (AOAC method 930.05), crude protein (AOAC method 978.04) using a nitrogen conversion factor of 6.25, total lipids (AOAC method 930.09), crude fibre (AOAC method 930.10), and total dietary fibre (AOAC methods 985.29 and 991.42 for insoluble dietary fibre), utilizing a Total Dietary Fiber Assay Kit (Megazyme, Ireland). Neutral detergent fibre (NDF), acid detergent fibre (ADF), and acid detergent lignin (ADL) were analysed using the Van Soest methodology to calculate cellulose, hemicellulose, and lignin, respectively [61]. Total carbon and total sulfur analysis were performed by oxidation at 1350 °C and infrared detection using a Leco SC-144 DR—Dual Range Sulfur and Carbon Analysis System (Leco, St Joseph, MI, USA). The total carbohydrates, available carbohydrates, and nitrogen-free extract were estimated following the guidelines of the Food and Agriculture Organization of the United Nations [62,63]. Energy values are expressed in kcal and kJ/100 g and were calculated according to Regulation (EU) n° 1169/2011 of the European Parliament and of the Council of 25 October 2011 [18].

### 3.3. Amino Acid Composition Analysis

The determination of the amino acid profile was performed by acid hydrolysis of the samples, followed by analysis using Waters^®^ Acquity UPLC system (Waters Corporation Company, Milford, MA, USA) equipped with photodiode array (PDA) detector (Waters Corporation Company, Milford, MA, USA), as described in [64].

### 3.4. Minerals Composition Analyses

The mineral content was analysed following the AOAC method 975.03 and quantified using flame atomic absorption spectrometry (FAAS) with a PerkinElmer PinAAcle 900 T Atomic Absorption Spectrometer (Waltham, MA, USA) [60]. Boron content was analysed using the SKALAR SAN + + System autoanalyzer (Skalar Analytical B.V., Breda, The Netherlands). Phosphorus levels, determined according to the AOAC method 948.09, were measured via spectrophotometry using a Hitachi U-2000 spectrophotometer (Hitachi, Tokyo, Japan) [60].

### 3.5. Fatty Acids Composition Analysis

The fatty acid composition of *D. muralis* leaves was determined as described in reference [65], by dissolving 0.5 g of lyophilized samples in 5 mL of heptane and converting them into methyl esters (FAMEs) through transesterification with the addition of 200 μL of 2 N sodium methoxide. Subsequently, 1 μL of the sample was analysed using a Chrompack CP 9001 gas chromatograph (Middelburg, The Netherlands) equipped with a flame ionization detector and a TR_CN 100 capillary column (60 m × 0.25 mm × 0.20 μm) (Teknokroma, Barcelona, Spain). Helium was used as the carrier gas. The injector and detector temperatures were set at 260 °C. The column oven program was as follows: the initial temperature was maintained at 60 °C for 7 min post-injection, then increased by 5 °C min^−1^ to 220 °C and held for an additional 15 min. Identification of fatty acids was conducted using an external authentic standard Supelco 37 Component FAME Mix (Supelco, PA, USA).

### 3.6. Food Safety

Lead and cadmium contents were analysed following the AOAC method 999.11 and quantified using graphite furnace atomic absorption spectrometry (GFAAS) with a PerkinElmer PinAAcle 900 T Atomic Absorption Spectrometer (Waltham, MA, USA) [66]. For mercury trace analysis, 0.15 g of sample was directly analysed by a Leco AMA254 Mercury Analyzer (St. Joseph, MI, USA).

For the establishment of *D. muralis* culture, soil analyses were conducted using the methodologies described in references [67,68]. Water analysis was performed using test kits and a DR3900 spectrophotometer (Hach, Loveland, CO, USA).

### 3.7. Extracts Preparation

For the extraction of bioactive compounds from the lyophilized plant material, different methods were employed [5]. A decoction was prepared by boiling 5 g of lyophilized plant material in 80% methanol with reflux for 6 h. Maceration was carried out using three different solvents: ethanol (100%), ethanol (50%), and water (100%). In each case, 5 g of lyophilized plant material was mixed with 100 mL of the respective solvent, followed by stirring for 6 h at room temperature. Additionally, an infusion was prepared by combining 5 g of lyophilized plant material with 100 mL of water, stirring it for 15 min. These methods allowed for the comparison of extraction efficiencies using different solvents and conditions. After filtration, the extract was concentrated using a rotary evaporator (Rotavapor R-114, Büchi, Flawil, Switzerland). The concentrated extracts were then lyophilized with a freeze-dryer (FTS Systems, Stone Ridge, NY, USA) and stored at −22 °C, protected from light and moisture.

### 3.8. Secondary Metabolites

#### 3.8.1. Total Phenolic Content

Total phenolic content (TPC) was determined using the Folin–Ciocalteu method [69]. To 0.5 mL of the sample, 0.5 mL of Folin–Ciocalteu reagent (Merck), 10 mL of 75 g L^−1^ sodium carbonate (Sigma-Aldrich, St. Louis, MI, USA), and water were added to reach a final volume of 25 mL. After 1 h of reaction, the absorbance was read at 750 nm using a spectrophotometer (U-2000 spectrophotometer, Hitachi, Tokyo, Japan). The analyses were performed in triplicates. The TPC was reported as mg gallic acid equivalent (GAE) per g.

#### 3.8.2. Total Flavonoids Content

Total flavonoids were determined using the aluminium chloride method [70]. The extract (1 mL) was mixed with 1 mL of 2% aluminium chloride (AlCl_3_) solution in methanol. The mixture was allowed to stand for 40 min, and its absorbance was measured at 415 nm using a spectrophotometer (U-2000 spectrophotometer, Hitachi, Tokyo, Japan). The analyses were performed in triplicate. The result was expressed in mg quercetin equivalent concentration (QE) per g.

#### 3.8.3. HPLC-PDA-ESI-MS^n^ Analysis

The phytochemical profile of *D. muralis* leaves after decoction in methanol (80%) was obtained using high-performance liquid chromatography (HPLC) (Finnigan Surveyor, Thermo, Waltham, MA, USA) coupled with a photodiode array (PDA) detector (Finnigan Surveyor, Thermo) and a linear ion trap mass spectrometer (LIT-MS) (LTQ XL, Thermo Scientific, Waltham, MA, USA). A Waters Spherisorb ODS2 C18 column (150 × 2.1 mm, 3 μm particle size) (Waters Corp., Milford, MA, USA) was used with mobile phases consisting of 5% (*v*/*v*) aqueous formic acid (solvent A) and acetonitrile (solvent B). The gradient conditions were as follows: 0 min, 0% B; 0–20 min, 10% B; 20–70 min, 100% B; and 70–90 min, 100% B. For the analysis, a concentration of 5 mg/mL was injected. UV–Visible spectra were acquired between 200 and 750 nm, and chromatographic profiles were recorded at wavelengths of 280 and 320 nm. MS spectra were acquired using negative and positive electrospray ionization (ESI) mass spectrometry. Helium was used as the collision gas with a collision energy of 35%. Nitrogen was used as the nebulizing gas with a sheath gas flow of 40 (arbitrary units) and as an auxiliary gas with a flow of 5 (arbitrary units). The temperature and voltage of the capillary were 275 °C and −35.00 V, respectively. The source voltage was 5.00 kV.

### 3.9. Antioxidant Activity

#### 3.9.1. 2,2′-Azinobis-(3-ethylbenzothiazoline-6-sulfonate) Assay (ABTS)

Total antioxidant capacity was determined by ABTS^•+^ method [69]. This method is based on ABTS^•+^ cationic radical colour loss measured as inhibition percentage after reading a spectrophotometer (U-2000 spectrophotometer, Hitachi, Tokyo, Japan) at 734 nm. The ABTS^•+^ solution was prepared by adding 1:1 (*v*/*v*) 7 mmol L^−1^ 2,2-azinobis(3-ethilbenzothyazoline-6-sulfonic acid diammonium salt (Sigma–Aldrich, St. Louis, MO, USA) to 2.45 mmol L^−1^ potassium persulfate (Merck, Germany) solutions. The reaction took place in the dark for 16 h and after the solution was diluted until an absorbance between 680–720 nm was achieved. 100 µL of the sample was used in order to obtain an inhibition percentage between 20 and 80%, by 6 min of reaction with 1 mL of ABTS^•+^ solution. The analyses were performed in triplicate. The result was expressed as mg ascorbic acid equivalent per g, mg trolox equivalent per g and IC_50_ (µg mL^−1^). Ascorbic acid and trolox were used as positive controls.

#### 3.9.2. 2,2-Diphenyl-1-picrylhydrazyl Radical Assay (DPPH)

The antiradical activities of plant extracts were determined using the free radical 2,2-diphenyl-1-picrylhydrazyl (DPPH·) [71]. The DPPH solution was prepared by dissolving 4.8 mg of DPPH^∙^ (Sigma–Aldrich, St. Louis, MO, USA) in 200 mL of methanol (Supelco, Merck, Germany). After adding 1.8 mL of the DPPH solution to 0.2 mL of the sample, the absorbance was measured after 15 min at 515 nm using a spectrophotometer (U-2000 spectrophotometer, Hitachi, Tokyo, Japan). The analyses were performed in triplicate. The result was expressed as mg trolox equivalent per g and IC_50_ (µg mL^−1^). Trolox was used as a positive control.

#### 3.9.3. Ferric Reducing Antioxidant Power Assay (FRAP)

The ferric reducing ability was evaluated by preparing the FRAP reagent, which was made by mixing 300 mM acetate buffer, 10 mL of TPTZ (Sigma–Aldrich, St. Louis, MO, USA) in 40 mM HCl, and 20 mM FeCl_3_.6H_2_O (Merck, Darmstadt, Germany) in the ratio of 10:1:1 (*v*/*v*/*v*) [72,73]. The extract (100 µL) was added to 3 mL of the FRAP reagent, and the absorbance was measured at 595 nm using a spectrophotometer (U-2000 spectrophotometer, Hitachi, Tokyo, Japan) after incubation at room temperature for 6 min. All determinations were performed in triplicate. The result was expressed as equivalent concentration of µmol Fe(II) equivalent per g and µmol trolox equivalent per g.

## 4. Conclusions

*D. muralis* shows an interesting nutritional profile, diverse bioactive compounds and strong antioxidant properties. The plant is rich in dietary fibre, essential minerals, amino acids, and fatty acids, with α-linolenic acid being notably predominant. Additionally, the levels of toxic metals were found to be below safety limits, ensuring the plant’s suitability for consumption.

Among the various extracts tested, the methanol decoction (80%) exhibited the highest concentrations of phenolic compounds and flavonoids, along with the strongest antioxidant activity. This extract was analysed using HPLC-PDA-ESI-MSn, revealing that quercetin-3-*O*-hexoside-dihexoside, quercetin-3,4′-diglucoside-3′-(6-sinapoyl-glucoside), and quercetin-3-*O*-deoxyhexose-hexose are the most abundant flavonols. For the first time in the *Diplotaxis* genus, the flavonoids quercetin-3-*O*-tetrahexoside, kaempferol-*O*-triglucoside, and isorhamnetin-*O*-trihexoside were identified. Of the five glucosinolates detected, glucoiberverin, glucopyranosyldisulfanyl-butyl-glucosinolate, and 6-methylsulfonyl-3-oxohexyl-glucosinolate stand out, as they have not been previously reported in this species. The identified glucosinolates and their isothiocyanates are responsible for the pungent and spicy flavours characteristic of *D. muralis*.

These results highlight its significant potential for health benefits and the development of functional foods. However, the quantification and identification of relevant secondary metabolites continue to present challenges, limiting a complete understanding of their contributions to bioactive properties. Further fractionation studies and detailed investigations into the factors influencing the composition and concentration of these compounds are crucial for a deeper understanding of their effects.

## Figures and Tables

**Table 1:** Nutritional profile of *D. muralis* leaves.

Composition	Raw Matter	Dry Matter
Moisture (g 100 g^−1^)	84.07 ± 0.02	-
Ash (g/100 g)	2.97 ± 0.01	18.67 ± 0.05
Total carbon (g 100 g^−1^)	4.89 ± 0.05	30.73 ± 0.30
Total sulfur (g 100 g^−1^)	0.15 ± 0.01	0.92 ± 0.03
Total nitrogen (g 100 g^−1^)	0.36 ± 0.00	2.28 ± 0.01
Protein (g 100 g^−1^)	2.27 ± 0.01	14.26 ± 0.06
Lipids (g 100 g^−1^)	0.53 ± 0.01	3.31 ± 0.09
Crude fibre (g 100 g^−1^)	1.46 ± 0.01	9.19 ± 0.07
Total dietary fibre (g 100 g^−1^)	4.70 ± 0.03	29.52 ± 0.19
Insoluble dietary fibre (g 100 g^−1^)	3.87 ± 0.02	24.28 ± 0.14
Soluble dietary fibre (g 100 g^−1^)	0.84 ± 0.01	5.25 ± 0.04
Neutral detergent fibre (g 100 g^−1^)	4.67 ± 0.03	29.33 ± 0.17
Acid detergent fibre (g 100 g^−1^)	2.69 ± 0.02	16.90 ± 0.14
Acid detergent lignin (g 100 g^−1^)	0.52 ± 0.01	3.25 ± 0.09
Cellulose (g 100 g^−1^)	1.61 ± 0.02	10.09 ± 0.14
Hemicellulose (g 100 g^−1^)	1.98 ± 0.01	12.42 ± 0.08
Lignin (g 100 g^−1^)	0.52 ± 0.01	3.25 ± 0.09
Available carbohydrates (g 100 g^−1^)	5.45 ± 0.03	34.24 ± 0.19
Nitrogen-free extract (g 100 g^−1^)	8.69 ± 0.02	54.57 ± 0.13
Total carbohydrates (includes fibre) (g 100 g^−1^)	10.15 ± 0.01	63.76 ± 0.06
Energy values (kcal/100 g)	45.04 ± 0.06	282.78 ± 0.35
Energy values (kJ/100 g)	188.56 ± 0.23	1183.96 ± 1.46

**Table 2:** Amino acid profile of *D. muralis* leaves.

Composition	Raw Matter	Dry Matter
Glutamic Acid (mg 100 g^−1^)	304.65 ± 14.60	1912.41 ± 91.64
Aspartic Acid (mg 100 g^−1^)	272.14 ± 6.05	1708.34 ± 37.99
Leucine (mg 100 g^−1^)	171.74 ± 9.54	1078.12 ± 59.88
Proline (mg 100 g^−1^)	150.14 ± 4.22	942.50 ± 26.49
Alanine (mg 100 g^−1^)	131.45 ± 4.81	825.18 ± 30.17
Lysine (mg 100 g^−1^)	125.78 ± 6.92	789.60 ± 43.43
Serine (mg 100 g^−1^)	114.68 ± 3.15	719.88 ± 19.75
Glycine (mg 100 g^−1^)	112.35 ± 3.99	705.26 ± 25.03
Valine (mg 100 g^−1^)	111.80 ± 5.75	701.81 ± 36.09
Phenylalanine (mg 100 g^−1^)	110.98 ± 7.13	696.69 ± 44.74
Arginine (mg 100 g^−1^)	94.36 ± 4.74	592.35 ± 29.76
Threonine (mg 100 g^−1^)	86.44 ± 4.92	542.59 ± 30.89
Tyrosine (mg 100 g^−1^)	84.94 ± 3.60	533.21 ± 22.58
Isoleucine (mg 100 g^−1^)	79.26 ± 4.43	497.54 ± 27.81
Histidine (mg 100 g^−1^)	46.13 ± 2.19	289.57 ± 13.74
Methionine (mg 100 g^−1^)	39.42 ± 3.13	247.47 ± 19.63
Cysteine (mg 100 g^−1^)	11.42 ± 0.18	71.72 ± 1.14

**Table 3:** Mineral content of *D. muralis* leaves.

Composition	Raw Matter	Dry Matter
Calcium (mg 100 g^−1^)	674.86 ± 8.88	4237.30 ± 55.77
Potassium (mg 100 g^−1^)	318.15 ± 8.84	1997.61 ± 55.53
Phosphorus (mg 100 g^−1^)	111.67 ± 0.23	701.16 ± 1.43
Magnesium (mg 100 g^−1^)	83.97 ± 3.09	527.21 ± 19.38
Sodium (mg 100 g^−1^)	21.48 ± 0.69	134.84 ± 4.35
Iron (mg 100 g^−1^)	3.88 ± 0.05	24.39 ± 0.29
Zinc (mg 100 g^−1^)	0.58 ± 0.02	3.63 ± 0.14
Manganese (mg 100 g^−1^)	0.46 ± 0.04	2.89 ± 0.24
Boron (mg 100 g^−1^)	0.30 ± 0.01	1.91 ± 0.09
Copper (mg 100 g^−1^)	0.11 ± 0.01	0.70 ± 0.06
Chromium (µg 100 g^−1^)	83.14 ± 3.16	522.02 ± 19.83
Nickel (µg 100 g^−1^)	60.19 ± 3.71	377.92 ± 23.27

**Table 4:** Fatty acids composition of *D. muralis* leaves expressed as relative percentages.

Fatty Acids Composition	*D. muralis* Leaves
Palmitic acid (C16:0)	14.36 ± 0.17
Margaric acid (C17:0)	8.03 ± 0.06
Stearic acid (C18:0)	0.85 ± 0.06
Oleic acid (C18:1)	11.41 ± 0.10
Linoleic acid (C18:2 n−6)	15.70 ± 0.10
α-Linolenic acid (C18:3 n−3)	40.58 ± 0.15
Arachidic acid (C20:0)	7.45 ± 0.07
Total	98.38 ± 0.06
SFA (saturated fatty acids)	30.69 ± 0.10
MUFA (monounsaturated fatty acids)	11.41 ± 0.10
PUFA (polyunsaturated fatty acids)	63.74 ± 0.18

**Table 5:** Heavy metal content of *D. muralis* leaves.

Composition	Raw Matter	Dry Matter
Lead (µg 100 g^−1^)	9.12 ± 0.07	57.25 ± 0.42
Cadmium (µg 100 g^−1^)	3.24 ± 0.02	20.37 ± 0.12
Mercury (µg 100 g^−1^)	1.06 ± 0.04	0.17 ± 0.01

**Table 6:** Results of total phenolic compounds, flavonoids in various extracts of *D. muralis*, expressed per mg of extract (mean ± standard deviation).

Type of Extract	Total Phenolic Compounds	Total Flavonoid Compounds
	mg eq. Gallic Acid g^−1^	mg eq. Quercetin g^−1^
Decoction methanol (80%)	68.36 ± 0.86	3.50 ± 0.05
Maceration ethanol (100%)	28.63 ± 0.36	1.76 ± 0.03
Maceration ethanol (50%)	15.92 ± 0.14	1.85 ± 0.04
Maceration water (100%)	11.16 ± 0.18	1.28 ± 0.03
Infusion water (100%)	9.44 ± 0.18	0.88 ± 0.02

**Table 7:** Compounds identified in the *D. muralis* leaves after decoction in methanol (80%) by HPLC-PDA-ESI-MS^n^.

Peak	Rt (min)	λmax (nm)	ESI-MS^n^ [*m*/*z* (Relative Abundance, %)]			Attempt to Identify [Reference]
			Precursor Ion [M−H]^−^	MS^2^	MS^3^	
1	1.60	-	333	333 (20); 241 (70); 153 (100)	153 (100); 97 (15); 79 (13)	Glycerophosphoinositol [23,24]
2	2.93	-	436	372 (100)	372 (100); 259 (95); 195 (45)	Glucoraphanin [25,26]
3	4.82	-	494	414 (100)	252 (100)	6-methylsulfonyl-3-oxohexyl- glucosinolate [27]
4	5.69	-	600	600 (100); 291 (73)	600(50); 404 (90); 291 (100)	Glucopyranosyldisulfanyl- butyl-glucosinolate [28]
5	9.00	-	420	420 (90); 259 (100)	259 (100); 139 (40)	Glucoerucin [26,29]
6	11.39	320	315	315 (100); 153 (100)	153 (100); 109 (35)	Gentisic acid-*O*-hexoside [30,31]
7	15.10	-	405	519 (100)	519 (70); 375 (60); 259 (100)	Glucoiberverin [29]
8	22.30	242; 266; 325	995 *	995 (100); 949 (70); 832 (90); 787 (100); 720 (70); 625 (65)	-	Quercetin-3-*O*-tetrahexoside [32]
9	24.97	249; 265; 320	817 *	771 (10); 609 (100); 447 (10)	447 (100)	Kaempferol-*O*-triglucoside
10	26.88	330	385	247 (40); 223 (100); 205 (30)	208 (70); 179 (40); 164 (100)	Sinapic acid-*O*-hexoside [33]
11	28.11	266; 335	833 *	787 (100)	625 (100); 463 (20); 301 (10)	Quercetin-3-*O*-hexoside- dihexoside
12	29.87	266; 322	609	609 (45); 447 (80); 285 (100)	285 (100); 151 (30)	Kaempferol-*O*-dihexoside [34]
13	30.10	266; 338	801	639 (100)	315 (100)	Isorhamnetin-*O*-trihexoside
14	30.26	266; 340	639	639 (50); 477 (80); 315 (100)	315 (100); 300 (90)	Isorhamnetin-*O*-dihexoside [34]
15	30.93	271; 328	993	831(100)	669 (100); 463 (30); 301 (5)	Quercetin-3,4′-diglucoside-3′-(6-sinapoyl-glucoside) [35]
16	31.42	255; 265sh; 353	609	609 (100); 301 (100)	301 (100); 179 (40); 151 (30)	Quercetin-3-*O*-deoxyhexose- hexose [34]
17	32.65	269; 318	593	593 (20); 285 (100)	285 (100)	Kaempferol-*O*-deoxyhesoside- hexoside [34]
18	33.04	252; 267sh; 330	639	315 (100)	315 (100); 300 (90)	Isorhamnetin-*O*-dihexoside [34]
19	34.03	326	753	529 (100)	511 (20); 299 (75); 223 (100); 205 (55)	Disinapoyl-diglucoside [36,37]
20	39.08	-	327	327 (30); 309 (20); 291 (55); 229 (100); 211 (30)	229 (90); 211 (100)	Oxo-dihydroxy-octadecenoic acid (DHODE) [38]
21	44.46	-	311	311 (100); 293(5)		Eicosanoic acid/Arachidic acid [38]
22	44.69	-	309	309 (100); 291 (5); 97(10)	309 (100); 97 (10)	8-hydroxy-9-oxo-octadecanoic acid [39]
23	45.12	-	311	311 (100); 293 (5)		Eicosanoic acid/Arachidic acid [38]
24	46.30	-	293	293(100); 275(5); 97 (20)	293 (100); 97 (20)	9-hydroxy-octadecatrienoic acid [38]
25	47.71	-	721 *	675 (100)	415 (20); 397 (100)	*O*-(Hexosyl-hexosyl)-*O*-linolenoyl-glycerol [40]

*: adduct; sh: shoulder; λmax.: maximum wavelength in UV-Vis spectrum.

**Table 8:** Antioxidant activity of various extracts from *D. muralis* (mean ± standard deviation).

Type of Extract	ABTS			DPPH		FRAP	
	mg eq. Ascorbic Acid g^−1^	mg eq. Trolox g^−1^	IC_50_ (µg mL^−1^)	mg eq. Trolox g^−1^	IC_50_ (µg mL^−1^)	µmol eq. Fe(II) g^−1^	µmol eq. Trolox g^−1^
Decoction methanol (80%)	30.14 ± 0.55	37.94 ± 0.12	78.87 ± 0.27	10.54 ± 0.01	392.95 ± 2.21	731.20 ± 5.41	259.80 ± 1.77
Maceration ethanol (100%)	8.73 ± 0.34	4.92 ± 0.65	181.39 ± 5.57	5.63 ± 0.03	547.72 ± 2.37	410.05 ± 3.65	145.47 ± 1.52
Maceration ethanol (50%)	15.84 ± 0.22	18.71 ± 0.42	112.14 ± 1.36	3.99 ± 0.07	711.94 ± 9.81	340.39 ± 1.31	117.18 ± 0.54
Maceration water (100%)	7.24 ± 0.13	5.43 ± 0.25	226.10 ± 3.21	2.60 ± 0.04	947.41 ± 8.52	174.23 ± 0.77	39.89 ± 0.32
Infusion water (100%)	7.57 ± 0.13	6.14 ± 0.25	218.69 ± 3.11	2.67 ± 0.03	958.59 ± 8.08	172.42 ± 0.64	54.01 ± 0.27
Control—Ascorbic acid	-	-	2.81 ± 0.02	-	-	-	-
Control—Trolox	-	-	2.25 ± 0.01	-	3.02 ± 0.02	-	-

## Data Availability

Data are contained within the article.

## Appendix A

Table A1 and Table A2 in Appendix A present the results of the soil and water analyses conducted prior to the establishment of the *D. muralis* crop.

**Table A1:** Soil analyses.

Parameters	Soil Composition
Field texture	Medium
Fine soil (Φ < 2 mm) (%)	86.85 ± 0.37
Organic matter (%)	3.9 ± 0.09
pH (H_2_O)	7.70 ± 0.02
Electrical conductivity (µS cm^−1^)	302.50 ± 2.38
Available phosphorus (mg P_2_O_5_ kg^−1^)	2009.03 ± 61.21
Available potassium (mg K_2_O kg^−1^)	203 ± 3.92
Available iron (mg Fe kg^−1^)	115 ± 1.97
Available copper (mg Cu kg^−1^)	54.6 ± 2.01
Available zinc (mg Zn kg^−1^)	47.5 ± 1.87
Available manganese (mg Mn kg^−1^)	34 ± 0.54
Available boron (mg B kg^−1^)	0.42 ± 0.04
Exchangeable cations—Potassium (meq. K^+^ 100 g^−1^)	0.47 ± 0.11
Exchangeable cations—Sodium (meq. Na^+^ 100 g^−1^)	0.08 ± 0.01
Exchangeable cations—Calcium (meq. Ca^2+^ 100 g^−1^)	10.40 ± 0.38
Exchangeable cations—Magnesium (meq. Mg^2+^ 100 g^−1^)	1.36 ± 0.25
Total Calcium (%)	4.22 ± 0.12
Total Potassium (%)	0.53 ± 0.05
Total Nitrogen (%)	0.25 ± 0.03
Total Phosphorus (%)	0.20 ± 0.04
Total Magnesium (%)	0.15 ± 0.02
Total Sodium (%)	0.14 ± 0.02
Total Zinc (mg Zn kg^−1^)	152.61 ± 1.01
Total Copper (mg Cu kg^−1^)	130.27 ± 1.18
Total Boron (mg B kg^−1^)	41.50 ± 1.06
Total Lead (mg Pb kg^−1^)	22.07 ± 0.67
Total Chromium (mg Cr kg^−1^)	10.05 ± 0.22
Total Nickel (mg Ni kg^−1^)	7.44 ± 0.11
Total Cadmium (mg Cd kg^−1^)	<1.03 (L.O.Q)
Total Mercury (mg Hg kg^−1^)	0.04 ± 0.00
L.O.Q: Limit of Quantification	

**Table A2:** Irrigation water analyses.

Parameters	Water Composition
pH (20 °C)	7.6 ± 0.0
Electrical conductivity (µS/cm, 20 °C)	465.2 ± 0.6
Total alkalinity mg/L (mg CaCO_3_ L^−1^)	26.6 ± 0.2
Oxidability (mg O_2_ L^−1^)	0.2 ± 0.0
Total hardness (mg CaCO_3_ L^−1^)	2.6 ± 0.1
Chlorides (mg L^−1^)	58.0 ± 0.3
Nitrates (mg L^−1^)	2.0 ± 0.1
Nitrites (mg L^−1^)	0.1 ± 0.0
Sulfates (mg L^−1^)	43.4 ± 0.2
Ammoniacal Nitrogen (mg L^−1^)	0.1 ± 0.0
Calcium (mg L^−1^)	18.6 ± 0.2
Iron (mg L^−1^)	0.2 ± 0.0
Magnesium (mg L^−1^)	15.0 ± 0.1
Potassium (mg L^−1^)	14.2 ± 0.1
Sodium (mg L^−1^)	8.2 ± 0.2

## References

[1] Pires A., Agreira S., Ressurreição S., Marques J., Guiné R., Barroca M.J., Moreira da Silva A. (2021). Sea Purslane as an Emerging Food Crop: Nutritional and Biological Studies. Appl. Sci..

[2] Milião G.L., de Oliveira A.P.H., Soares L.d.S., Arruda T.R., Vieira É.N.R., Leite Junior B.R.d.C. (2022). Unconventional Food Plants: Nutritional Aspects and Perspectives for Industrial Applications. Future Foods.

[3] Alae-Carew C., Green R., Stewart C., Cook B., Dangour A.D., Scheelbeek P.F.D. (2022). The Role of Plant-Based Alternative Foods in Sustainable and Healthy Food Systems: Consumption Trends in the UK. Sci. Total Environ..

[4] Sabaté J., Soret S. (2014). Sustainability of Plant-Based Diets: Back to the Future123. Am. J. Clin. Nutr..

[5] Ressurreição S., Salgueiro L., Figueirinha A. (2024). Diplotaxis Genus: A Promising Source of Compounds with Nutritional and Biological Properties. Molecules.

[6] Caruso G., Parrella G., Giorgini M., Nicoletti R. (2018). Crop Systems, Quality and Protection of *Diplotaxis tenuifolia*. Agriculture.

[7] Bell L., Oloyede O.O., Lignou S., Wagstaff C., Methven L. (2018). Taste and Flavor Perceptions of Glucosinolates, Isothiocyanates, and Related Compounds. Mol. Nutr. Food Res..

[8] Pasini F., Verardo V., Cerretani L., Caboni M.F., D’Antuono L.F. (2011). Rocket Salad (Diplotaxis and Eruca Spp.) Sensory Analysis and Relation with Glucosinolate and Phenolic Content. J. Sci. Food Agric..

[9] Fukalova T., García Martínez M.D., Raigón M.D. (2021). Five Undervalued Edible Species Inherent to Autumn-Winter Season: Nutritional Composition, Bioactive Constituents and Volatiles Profile. PeerJ.

[10] Jdir H., Khemakham B., Chakroun M., Zouari S., Ali Y.B., Zouari N. (2015). *Diplotaxis simplex* Suppresses Postprandial Hyperglycemia in Mice by Inhibiting Key-Enzymes Linked to Type 2 Diabetes. Rev. Bras. De Farmacogn..

[11] Pimpini F., Giannini M., Lazzarin R. (2005). Ortaggi Da Foglia e Da Taglio.

[12] Instituto Nacional de Saúde Doutor Ricardo Jorge (INSA) Plataforma Portuguesa de Informação Alimentar (PortFIR). https://jspapp.test.insa.foodcase-services.com/.

[13] Guan Z.-W., Yu E.-Z., Feng Q. (2021). Soluble Dietary Fiber, One of the Most Important Nutrients for the Gut Microbiota. Molecules.

[14] Lisiewska Z., Kmiecik W., Korus A. (2008). The Amino Acid Composition of Kale (*Brassica oleracea* L. Var. *Acephala*), Fresh and after Culinary and Technological Processing. Food Chem..

[15] Bell L., Methven L., Signore A., Oruna-Concha M.J., Wagstaff C. (2017). Analysis of Seven Salad Rocket (*Eruca sativa*) Accessions: The Relationships between Sensory Attributes and Volatile and Non-Volatile Compounds. Food Chem..

[16] Murcia M.A., López-Ayerra B., Martínez-Tomé M., García-Carmona F. (2001). Effect of Industrial Processing on Amino Acid Content of Broccoli. J. Sci. Food Agric..

[17] Martínez S., Losada P., Franco I., Carballo J. (2011). Protein, Amino Acid, Ash and Mineral Contents in Brassica Spp. Grown in Northwest Spain. Int. J. Food Sci. Technol..

[18] Official Journal of the European Union (2011). Regulation (EU) No 1169/2011 of the European Parliament and of the Council of 25 October 2011 on the Provision of Food Information to Consumers (Text with EEA Relevance).

[19] Gisbert C., Clemente R., Navarro-Aviñó J., Baixauli C., Ginér A., Serrano R., Walker D.J., Bernal M.P. (2006). Tolerance and Accumulation of Heavy Metals by Brassicaceae Species Grown in Contaminated Soils from Mediterranean Regions of Spain. Environ. Exp. Bot..

[20] Official Journal of the European Union (2023). Commission Regulation (EU) 2023/915 of 25 April 2023 on Maximum Levels for Certain Contaminants in Food and Repealing Regulation (EC) No 1881/2006 (Text with EEA Relevance).

[21] Falleh H., Msilini N., Oueslati S., Ksouri R., Magne C., Lachaâl M., Karray-Bouraoui N. (2013). *Diplotaxis Harra* and *Diplotaxis Simplex* Organs: Assessment of Phenolics and Biological Activities before and after Fractionation. Ind. Crops Prod..

[22] Ahmed A.F., Wen Z.-H., Bakheit A.H., Basudan O.A., Ghabbour H.A., Al-Ahmari A., Feng C.-W. (2022). A Major Diplotaxis Harra-Derived Bioflavonoid Glycoside as a Protective Agent against Chemically Induced Neurotoxicity and Parkinson’s Models; In Silico Target Prediction; and Biphasic HPTLC-Based Quantification. Plants.

[23] Ağalar H.G., Akalın Çiftçi G., Göger F., Kırımer N. (2017). Activity Guided Fractionation of Arum Italicum Miller Tubers and the LC/MS-MS Profiles. Rec. Nat. Prod..

[24] Grauso L., Mariggiò S., Corda D., Fontana A., Cutignano A. (2015). An Improved UPLC-MS/MS Platform for Quantitative Analysis of Glycerophosphoinositol in Mammalian Cells. PLoS ONE.

[25] Fabre N., Poinsot V., Debrauwer L., Vigor C., Tulliez J., Fourasté I., Moulis C. (2007). Characterisation of Glucosinolates Using Electrospray Ion Trap and Electrospray Quadrupole Time-of-Flight Mass Spectrometry. Phytochem. Anal..

[26] Dong M., Tian Z., Ma Y., Yang Z., Ma Z., Wang X., Li Y., Jiang H. (2021). Rapid Screening and Characterization of Glucosinolates in 25 Brassicaceae Tissues by UHPLC-Q-Exactive Orbitrap-MS. Food Chem..

[27] Cataldi T.R.I., Lelario F., Orlando D., Bufo S.A. (2010). Collision-Induced Dissociation of the A + 2 Isotope Ion Facilitates Glucosinolates Structure Elucidation by Electrospray Ionization-Tandem Mass Spectrometry with a Linear Quadrupole Ion Trap. Anal. Chem..

[28] Cataldi T.R.I., Rubino A., Lelario F., Bufo S.A. (2007). Naturally Occurring Glucosinolates in Plant Extracts of Rocket Salad (*Eruca sativa* L.) Identified by Liquid Chromatography Coupled with Negative Ion Electrospray Ionization and Quadrupole Ion-Trap Mass Spectrometry. Rapid Commun. Mass Spectrom..

[29] D’Antuono L.F., Elementi S., Neri R. (2008). Glucosinolates in Diplotaxis and Eruca Leaves: Diversity, Taxonomic Relations and Applied Aspects. Phytochemistry.

[30] Grati W., Samet S., Bouzayani B., Ayachi A., Treilhou M., Téné N., Mezghani-Jarraya R. (2022). HESI-MS/MS Analysis of Phenolic Compounds from Calendula Aegyptiaca Fruits Extracts and Evaluation of Their Antioxidant Activities. Molecules.

[31] Robbins R.J. (2003). Phenolic Acids in Foods:  An Overview of Analytical Methodology. J. Agric. Food Chem..

[32] Ferreres F., Fernandes F., Oliveira J.M.A., Valentão P., Pereira J.A., Andrade P.B. (2009). Metabolic Profiling and Biological Capacity of *Pieris Brassicae* Fed with Kale (*Brassica oleracea* L. Var. *Acephala*). Food Chem. Toxicol..

[33] Spínola V., Pinto J., Castilho P.C. (2015). Identification and Quantification of Phenolic Compounds of Selected Fruits from Madeira Island by HPLC-DAD–ESI-MS*n* and Screening for Their Antioxidant Activity. Food Chem..

[34] Loizzo M.R., Napolitano A., Bruno M., Geraci A., Schicchi R., Leporini M., Tundis R., Piacente S. (2021). LC-ESI/HRMS Analysis of Glucosinolates, Oxylipins and Phenols in Italian Rocket Salad (*Diplotaxis erucoides* Subsp. *erucoides* (L.) DC.) and Evaluation of Its Healthy Potential. J. Sci. Food Agric..

[35] Bell L., Oruna-Concha M.J., Wagstaff C. (2015). Identification and Quantification of Glucosinolate and Flavonol Compounds in Rocket Salad (*Eruca sativa*, *Eruca vesicaria* and *Diplotaxis tenuifolia*) by LC–MS: Highlighting the Potential for Improving Nutritional Value of Rocket Crops. Food Chem..

[36] Sun J., Xiao Z., Lin L., Lester G.E., Wang Q., Harnly J.M., Chen P. (2013). Profiling Polyphenols in Five Brassica Species Microgreens by UHPLC-PDA-ESI/HRMSn. J. Agric. Food Chem..

[37] Olsen H., Aaby K., Borge G.I.A. (2009). Characterization and Quantification of Flavonoids and Hydroxycinnamic Acids in Curly Kale (*Brassica oleracea* L. Convar. *acephala* Var. *sabellica*) by HPLC-DAD-ESI-MSn. J. Agric. Food Chem..

[38] Islam A.K.M.M., Hong S.-M., Lee H.-S., Moon B.-C., Kim D., Kwon H. (2018). Identification and Characterization of Matrix Components in Spinach during QuEChERS Sample Preparation for Pesticide Residue Analysis by LC–ESI–MS/MS, GC–MS and UPLC-DAD. J. Food Sci. Technol..

[39] Oliw E.H., Aragó M., Chen Y., Jernerén F. (2016). A new class of fatty acid allene oxide formed by the DOX-P450 fusion proteins of human and plant pathogenic fungi, *C. immitis* and *Z. tritici*. J. Lipid Res..

[40] Abdul Khaliq H., Ortiz S., Alhouayek M., Muccioli G.G., Quetin-Leclercq J. (2022). Dereplication and Quantification of Major Compounds of *Convolvulus arvensis* L. Extracts and Assessment of Their Effect on LPS-Activated J774 Macrophages. Molecules.

[41] Melrose J. (2019). The Glucosinolates: A Sulphur Glucoside Family of Mustard Anti-Tumour and Antimicrobial Phytochemicals of Potential Therapeutic Application. Biomedicines.

[42] Jdir H., Elfalleh W., Najjaa H., Jridi M., Abousalham A., Zouari N., Fakhfakh N. (2020). Phenolic Compounds from the Cruciferous Diplotaxis Simplex and Their Inhibitory Effects on Pancreatic Lipase and Cancer Cells. Experiment.

[43] Jdir H., Jridi M., Mabrouk M., Ayadi M.A., Nasri M., Zouari N., Fakhfakh N. (2017). The Rocket, *Diplotaxis Simplex*, as a Functional Ingredient: LC-ESI-MS Analysis and Its Effect on Antioxidant and Physical Properties of Bread. J. Food Nutr. Res..

[44] Jiang C., Gates P.J. (2024). Systematic Characterisation of the Fragmentation of Flavonoids Using High-Resolution Accurate Mass Electrospray Tandem Mass Spectrometry. Molecules.

[45] Bell L., Wagstaff C. (2014). Glucosinolates, Myrosinase Hydrolysis Products, and Flavonols Found in Rocket (*Eruca sativa* and *Diplotaxis tenuifolia*). J. Agric. Food Chem..

[46] Martínez-Sánchez A., Llorach R., Gil M.I., Ferreres F. (2007). Identification of New Flavonoid Glycosides and Flavonoid Profiles To Characterize Rocket Leafy Salads (*Eruca vesicaria* and *Diplotaxis tenuifolia*). J. Agric. Food Chem..

[47] Pasini F., Verardo V., Caboni M.F., D’Antuono L.F. (2012). Determination of Glucosinolates and Phenolic Compounds in Rocket Salad by HPLC-DAD–MS: Evaluation of *Eruca sativa* Mill. and *Diplotaxis tenuifolia* L. Genetic Resources. Food Chem..

[48] Bennett R.N., Rosa E.A.S., Mellon F.A., Kroon P.A. (2006). Ontogenic Profiling of Glucosinolates, Flavonoids, and Other Secondary Metabolites in *Eruca sativa* (Salad Rocket), *Diplotaxis erucoides* (Wall Rocket), *Diplotaxis tenuifolia* (Wild Rocket), and *Bunias orientalis* (Turkish Rocket). J. Agric. Food Chem..

[49] Bahloul N., Bellili S., Aazza S., Chérif A., Faleiro M.L., Antunes M.D., Miguel M.G., Mnif W. (2016). Aqueous Extracts from Tunisian Diplotaxis: Phenol Content, Antioxidant and Anti-Acetylcholinesterase Activities, and Impact of Exposure to Simulated Gastrointestinal Fluids. Antioxidants.

[50] Scalbert A., Johnson I.T., Saltmarsh M. (2005). Polyphenols: Antioxidants and Beyond. Am. J. Clin. Nutr..

[51] González R., Ballester I., López-Posadas R., Suárez M.D., Zarzuelo A., Martínez-Augustin O., Medina F.S.D. (2011). Effects of Flavonoids and Other Polyphenols on Inflammation. Crit. Rev. Food Sci. Nutr..

[52] Fahey J.W., Zalcmann A.T., Talalay P. (2001). The Chemical Diversity and Distribution of Glucosinolates and Isothiocyanates among Plants. Phytochemistry.

[53] Nugrahedi P.Y., Verkerk R., Widianarko B., Dekker M. (2015). A Mechanistic Perspective on Process-Induced Changes in Glucosinolate Content in Brassica Vegetables: A Review. Crit. Rev. Food Sci. Nutr..

[54] Coutinho L.d.L., Junior T.C.T., Rangel M.C. (2023). Sulforaphane: An Emergent Anti-Cancer Stem Cell Agent. Front. Oncol..

[55] Asif Ali M., Khan N., Kaleem N., Ahmad W., Alharethi S.H., Alharbi B., Alhassan H.H., Al-Enazi M.M., Razis A.F.A., Modu B. (2023). Anticancer Properties of Sulforaphane: Current Insights at the Molecular Level. Front. Oncol..

[56] Melchini A., Costa C., Traka M., Miceli N., Mithen R., De Pasquale R., Trovato A. (2009). Erucin, a New Promising Cancer Chemopreventive Agent from Rocket Salads, Shows Anti-Proliferative Activity on Human Lung Carcinoma A549 Cells. Food Chem. Toxicol..

[57] Guerreiro Í., Vidovic B., Costa J.G., Martins M., Ferreira S., Oliveira N.G., Saraiva N., Fernandes A.S. (2023). The Dietary Isothiocyanate Erucin Reduces Kidney Cell Motility by Disturbing Tubulin Polymerization. Mol. Nutr. Food Res..

[58] Calder P.C. (2017). Omega-3 Fatty Acids and Inflammatory Processes: From Molecules to Man. Biochem. Soc. Trans..

[59] Serhan C.N., Chiang N., Van Dyke T.E. (2008). Resolving Inflammation: Dual Anti-Inflammatory and pro-Resolution Lipid Mediators. Nat. Rev. Immunol..

[60] Cunniff P. (1997). Official Methods of Analysis of AOAC International.

[61] Van Soest P.J., Robertson J.B., Lewis B.A. (1991). Methods for Dietary Fiber, Neutral Detergent Fiber, and Nonstarch Polysaccharides in Relation to Animal Nutrition. J. Dairy Sci..

[62] Food and Agriculture Organization of the United Nations (2003). Food Energy—Methods of Analysis and Conversion Factors.

[63] Tacon A.G.J. (1987). The Nutrition and Feeding of Farmed Fish and Shrimp—A Training Manual—2. Nutrient Sources and Composition.

[64] Mota C., Santos M., Mauro R., Samman N., Matos A.S., Torres D., Castanheira I. (2016). Protein Content and Amino Acids Profile of Pseudocereals. Food Chem..

[65] Assunção M.F.G., Varejão J.M.T.B., Santos L.M.A. (2017). Nutritional Characterization of the Microalga *Ruttnera Lamellosa* Compared to *Porphyridium Purpureum*. Algal Res..

[66] AOAC International (2002). Official Methods of Analysis of AOAC International.

[67] Ferreira C.S., Veiga A., Caetano A., Gonzalez-Pelayo O., Karine-Boulet A., Abrantes N., Keizer J., Ferreira A.J. (2020). Assessment of the Impact of Distinct Vineyard Management Practices on Soil Physico-Chemical Properties. Air Soil Water Res..

[68] Clescari L.S., Greenberg A.E., Eaton A.D. (1998). Standard Methods for the Examination of Water and Wastewater.

[69] Gião M.S., González-Sanjosé M.L., Rivero-Pérez M.D., Pereira C.I., Pintado M.E., Malcata F.X. (2007). Infusions of Portuguese Medicinal Plants: Dependence of Final Antioxidant Capacity and Phenol Content on Extraction Features. J. Sci. Food Agric..

[70] Al-Dabbas M.M., Suganuma T., Kitahara K., Hou D.-X., Fujii M. (2006). Cytotoxic, Antioxidant and Antibacterial Activities of *Varthemia Iphionoides* Boiss. Extracts. J. Ethnopharmacol..

[71] Brand-Williams W., Cuvelier M.E., Berset C. (1995). Use of a Free Radical Method to Evaluate Antioxidant Activity. LWT Food Sci. Technol..

[72] Pulido R., Bravo L., Saura-Calixto F. (2000). Antioxidant Activity of Dietary Polyphenols As Determined by a Modified Ferric Reducing/Antioxidant Power Assay. J. Agric. Food Chem..

[73] Pedreiro S., da Ressurreição S., Lopes M., Cruz M.T., Batista T., Figueirinha A., Ramos F. (2021). *Crepis vesicaria* L. Subsp. Taraxacifolia Leaves: Nutritional Profile, Phenolic Composition and Biological Properties. IJERPH.

